# Competitive Interactions Between Incompatible Mutants of the Social Bacterium *Myxococcus xanthus* DK1622

**DOI:** 10.3389/fmicb.2018.01200

**Published:** 2018-06-05

**Authors:** Ya Gong, Zheng Zhang, Xiu-wen Zhou, Mian N. Anwar, Xiao-zhuang Hu, Ze-shuo Li, Xiao-jing Chen, Yue-zhong Li

**Affiliations:** State Key Laboratory of Microbial Technology, School of Life Science, Shandong University, Jinan, China

**Keywords:** *Myxococcus xanthus*, transposon-mutation, multiple genetic loci, self-identification, competitive interaction, colony-merger incompatibility

## Abstract

Due to the high similarity in their requirements for space and food, close bacterial relatives may be each other's strongest competitors. Close bacterial relatives often form visible boundaries to separate their swarming colonies, a phenomenon termed colony-merger incompatibility. While bacterial species are known to have many incompatible strains, it is largely unclear which traits lead to multiple incompatibilities and the interactions between multiple incompatible siblings. To investigate the competitive interactions of closely related incompatible strains, we mutated *Myxococcus xanthus* DK1622, a predatory bacterium with complex social behavior. From 3392 random transposon mutations, we obtained 11 self-identification (SI) deficient mutants that formed unmerged colony boundaries with the ancestral strain. The mutations were at nine loci with unknown functions and formed nine independent SI mutants. Compared with their ancestral strain, most of the SI mutants showed reduced growth, swarming and development abilities, but some remained unchanged from their monocultures. When pairwise mixed with their ancestral strain for co-cultivation, these mutants exhibited improved, reduced or unchanged competitive abilities compared with the ancestral strain. The sporulation efficiencies were affected by the DK1622 partner, ranging from almost complete inhibition to 360% stimulation. The differences in competitive growth between the SI mutants and DK1622 were highly correlated with the differences in their sporulation efficiencies. However, the competitive efficiencies of the mutants in mixture were inconsistent with their growth or sporulation abilities in monocultures. We propose that the colony-merger incompatibility in *M. xanthus* is associated with multiple independent genetic loci, and the incompatible strains hold competitive interaction abilities, which probably determine the complex relationships between multiple incompatible *M. xanthus* strains and their co-existence strategies.

## Introduction

Hamilton's kin selection rule asserts that the closer the kin relationship between individuals, the stronger their cooperative tendencies and altruistic behavior (Hamilton, 1964; West et al., 2006; Strassmann et al., 2011; Waibel et al., 2011). However, because of their highly similar requirements for space and food, close relatives are potentially the strongest competitive neighbors in nature. Kin discrimination provides a means for individuals to identify self and non-self, thus separating competitive neighbors and probably aiding the survival of different kin groups. Closely related strains represent the basic units of bacterial communities in nature (Hamilton, 1964; West et al., 2006; Strassmann et al., 2011; Waibel et al., 2011). Many bacterial cells are able to discriminate self from non-self relatives by forming boundaries between encountered colonies (Dienes, 1946; Munson et al., 2002; Gibbs et al., 2008; Vos and Velicer, 2009; Rendueles et al., 2015; Stefanic et al., 2015; Lyons et al., 2016). Colony-merger incompatibility is a type of bacterial kin discrimination that results in the separation of different groups, and probably allows the existence of diverse incompatible strains within bacterial species in a small patch of soil. For example, a centimeter-scale soil sample yielded 45 incompatible types when 78 *Myxococcus xanthus* isolates were pairwise inoculated in close proximity on growth medium (Vos and Velicer, 2009). Similarly, among 39 isolates of *Bacillus subtilis* from two 1-cm^3^ soil samples, 12 incompatible types were identified with striking boundaries between their swarms (Stefanic et al., 2015). The incompatible characteristics and the interactions between incompatible siblings are suggested to be important for understanding the co-existence mechanisms of incompatible groups and their ecological functions (Griffin et al., 2004; Keller and Surette, 2006; West et al., 2007; Brown and Buckling, 2008; Velicer and Vos, 2009; Li et al., 2013). Gibbs and Greenberg proposed that the boundaries formed between incompatible bacterial strains can establish and harbor an area in which one strain occupies the space and food exclusively and protects itself against colonization by a competing strain (Gibbs and Greenberg, 2011). Such a colony boundary is also suggested to be a barrier to homologous recombination (gene flow) between closely related incompatible strains, which may promote ecological divergence and co-existence (Wielgoss et al., 2016). Natural incompatible sibling isolates normally have high mutation frequencies and horizontal gene transfers, which make the comparative genomics of natural isolates difficult to interpret with regard to the incompatibility of phenotypes. Incompatible laboratory mutants derived from the same ancestral strains can contribute to our understanding of the incompatibility characteristics and the interactions between incompatible siblings, and also facilitate further research.

*Myxococcus xanthus* displays complex social behavior (Shimkets, 1990; Dworkin, 1996). *M. xanthus* cells locomote on solid surfaces in swarms and prey collaboratively on other microbial cells in a wolf-pack pattern. When food is scarce, the *M. xanthus* cells aggregate to develop multicellular fruiting bodies, inside which stress-resistant myxospores are formed. Social behavior occurs between the cells of a single *Myxococcus* strain. If two different *Myxococcus* species or strains of the same species are co-cultured, they might occupy separate territories (Smith and Dworkin, 1994; Vos and Velicer, 2009). The number of incompatible strains—for example, 45 incompatible types in the 78 *M. xanthus* centimeter-scale isolates (Vos and Velicer, 2009)—suggests that closely related incompatible siblings may possess various features and their interactions are entirely competitive. However, these previous studies were performed with *Myxococcus* natural strains with unclear genetic backgrounds, leaving the underlying mechanisms unclear. In this study, to investigate the traits leading to the multiple incompatibilities and the competitive interactions between multiple incompatible siblings, we mutated *M. xanthus* DK1622, a model myxobacterium strain, by random transposon insertion mutagenesis and obtained multiple colony-merger incompatible mutants. We checked the cellular growth abilities and social characteristics of these self-identification (SI) mutants in monocultures to determine their relevance to the colony-merger incompatibility. We assayed their competitive interactions in co-cultures during growth and sporulation and found that the mutants exhibited improved, reduced or unchanged competitive abilities in mixture; however, these abilities were not relevant to their growth or development abilities in monoculture. We determined that multiple colony-merger incompatibilities in *M. xanthus* are associated with different genetic loci and interactions between the incompatible strains are diverse, reflecting the diversity of the underlying mechanisms of incompatibility. We discuss the significance of competitive interactions between co-existing incompatible close relatives in nature.

## Results

### Colony-merger incompatibility mutations in *Myxococcus xanthus* DK1622

**Figure 1:**
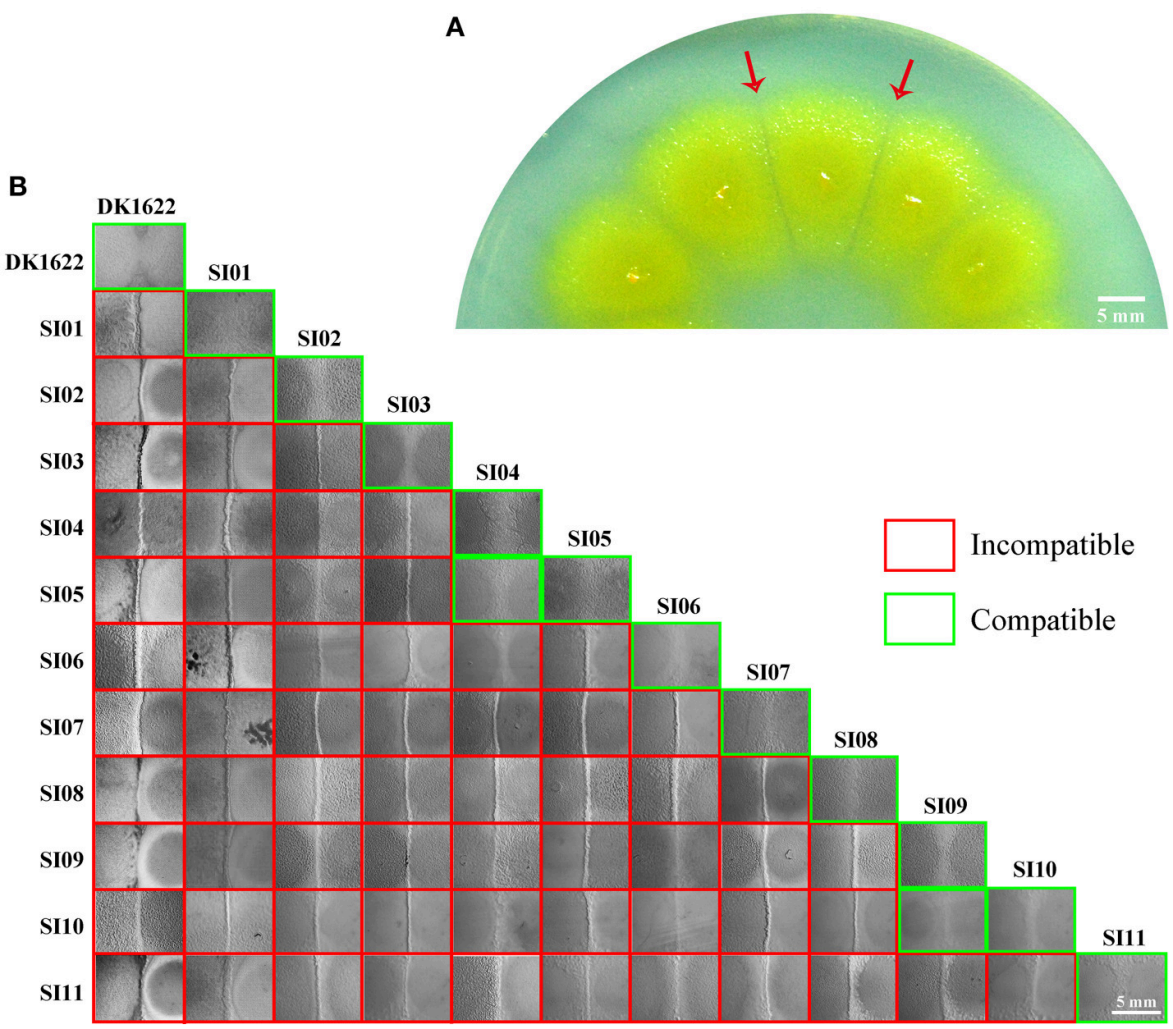
Screening for incompatible mutants in *M. xanthus* DK1622. **(A)** Visible boundaries (red arrows) formed between two adjacent colonies of pMiniHimar-*lacZ* insertion mutants on CTT agar plates supplemented with kanamycin. **(B)** The colony development between the 11 SI mutants and DK1622. All photos are representative of three biological replicates. Scale bar, 5 mm **(A,B)**.

We made insertion mutations in *M. xanthus* DK1622 using the pMiniHimar-*lacZ* plasmid, which is able to insert into genomes randomly (Rubin et al., 1999; Youderian et al., 2003). To screen for incompatible mutants that formed visible colony boundaries with neighbors, transformants were inoculated adjacent to each other on CTT growth medium (Hodgkin and Kaiser, 1977) supplemented with kanamycin. Figure 1A shows an incompatible mutant with its neighboring mutants. The incompatible kanamycin-resistant recombinants were further inoculated adjacent to the wild-type strain on a CTT plate to confirm their colony-merger incompatibilities. From 3,392 mutations, we obtained 11 mutants that formed visible colony boundaries with DK1622, while two colonies of DK1622 or the same mutant strain were merged (Figure 1B). Southern blotting experiments indicated that each of the 11 mutants contained a single insertion in the genome. We named these incompatible mutants as SI mutants SI01–SI11. We examined the boundary formation between the incompatible strains under a microscope. In two adjacently inoculated incompatible colonies, the cells that moved toward the opposite colonies stopped moving forward when encountered. The accumulated cells protruded vertically at the colony edges, thus forming a boundary ravine separating the two colonies. Video S1 demonstrates the boundary formation between the SI01 mutant and the wild-type DK1622. Furthermore, a dyeing experiment showed that the colony boundaries between SI mutants and the wild-type strain might contain damaged cells (Figure S1 demonstrates the dyeing result of the adjacent colonies of DK1622 and SI04).

Using plasmid-rescuing techniques, we located the insertion sites of the SI mutants. Sequencing revealed that the 11 mutations were at 9 genetic loci scattered throughout the genome of *M. xanthus* DK1622 (NC_008095.1; Table 1). All of the 11 mutations were located in open reading frames, including three newly annotated genes (*MXAN_RS36575* in SI05, *MXAN_RS24590* in SI08 and *MXAN_RS34540* in SI10). Three of these genes were annotated to encode a putative regulator of ribonuclease A (*MXAN_0390*, SI03; Goldman et al., 2006), a putative methyltransferase (*MXAN_1599*, SI06) and a serine/threonine protein kinase (*MXAN_7251*, SI11), while the others were hypothetical open reading frames. These mutated genes have not yet been studied genetically or biochemically. We further tried to delete each of the insertion-mutated genes in the wild-type strain DK1622, and six deletion mutants were obtained and exhibited similar incompatibilities to the insertion mutants; i.e., the deletion mutants merged their colonies with the corresponding insertion mutants, but formed colony boundaries with the wild-type strain (Figure S2). The six genes were *MXAN_0049* in SI01, *MXAN_0085* in SI02, *MXAN_RS36575* in SI05, *MXAN_2099* in SI07, *MXAN_RS24590* in SI08 and *MXAN_RS34540* in SI10, suggesting that these six genes were responsible for the colony-merger incompatibility.

**Table 1:** Eleven SI mutants and their information.

**Mutants**	**Locus inserted**	**Length (bp)**	**Putative identification**
SI01	*MXAN_0049*	585	hypothetical protein
SI02	*MXAN_0085*	588	hypothetical protein
SI03	*MXAN_0390*	498	regulator of ribonuclease activity A, putative
SI04	*MXAN_1307*	867	hypothetical protein
SI05	*MXAN_RS36575* (between *MXAN_1307* and *MXAN_1308*)	585	hypothetical protein
SI06	*MXAN_1599*	1,500	putative methyltransferase
SI07	*MXAN_2099*	561	conserved hypothetical protein
SI08	*MXAN_RS24590* (between *MXAN_5062* and *MXAN_5063*)	462	hypothetical protein
SI09	*MXAN_7134*	777	hypothetical protein
SI10	*MXAN_RS34540* (between *MXAN_7134* and *MXAN_7135*)	588	hypothetical protein
SI11	*MXAN_7251*	3,627	serine/threonine protein kinase

When these mutants were pairwise inoculated adjacent to each other on a CTT plate, two pairs (SI04/SI05 and SI09/SI10) formed merged colonies, while the other pairs were separated from each other by an obvious colony boundary (Figure 1B). Notably, the two pairs that merged colonies had close insertion sites, consistent with their similarly compatible phenotypes. The insertion site in the SI04 mutant was in *MXAN_1307*, while the mutation in SI05 was in a new gene upstream to *MXAN_1307*. Similarly, the mutation in the SI09 mutant was located in *MXAN_7134*, while the SI10 mutant was inserted in a newly annotated gene upstream to *MXAN_7134* (Table 1). Thus, the 11 SI mutants formed 9 independent incompatible mutants that were derived from 9 different genetic determinants. Because the nine insertion-mutated genetic loci were scattered throughout the genome of *M. xanthus* (Table 1), we propose that the nine independent genetic determinants might be involved in colony-merger incompatibility.

### The SI mutants showed variable growth abilities and retained multicellular social behavior

**Figure 2:**
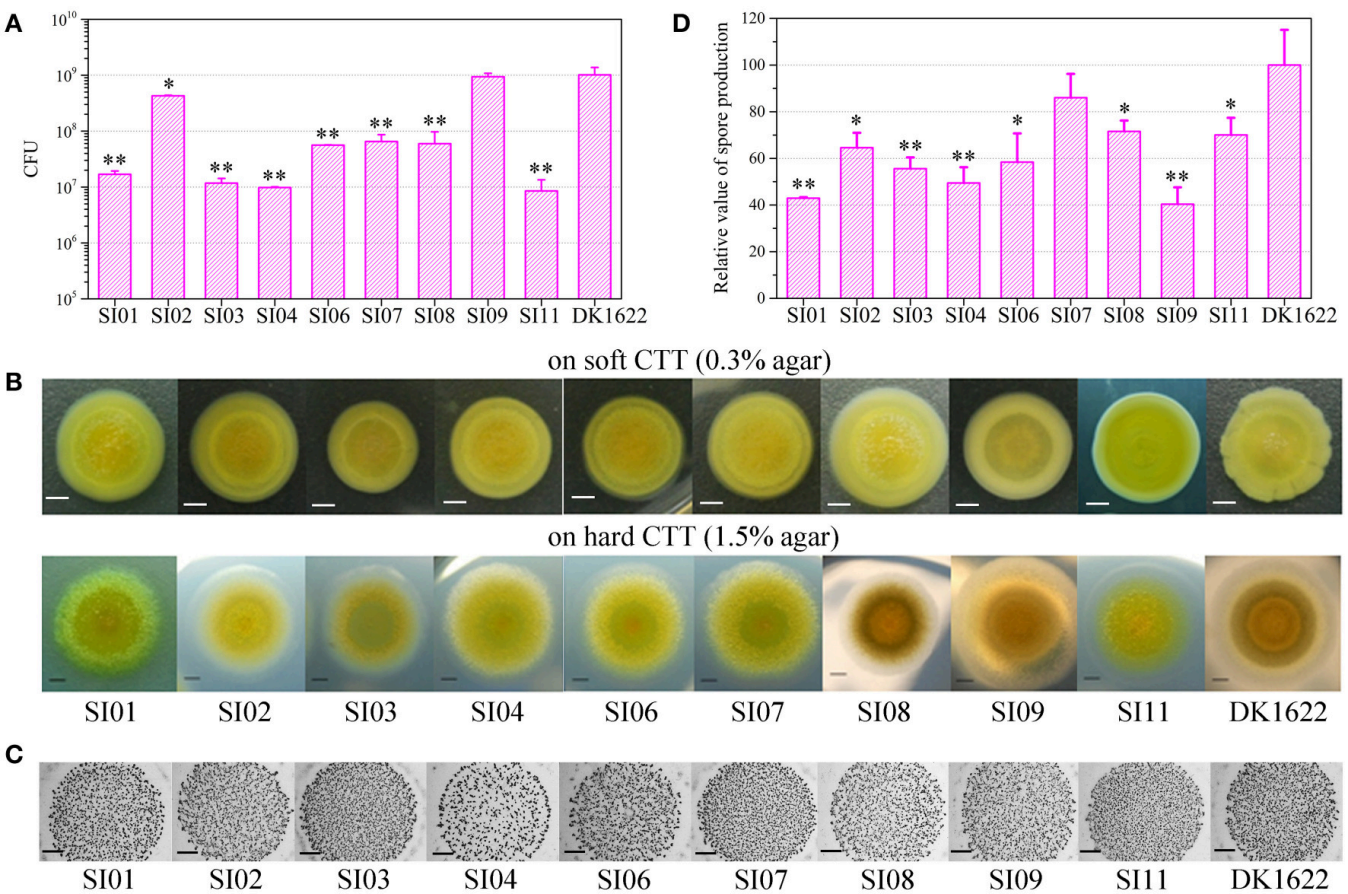
Characteristics of incompatible mutants. **(A)** Colony-forming unit (CFU) counts of incompatible mutants and DK1622 after incubation for 48 hr on CTT medium. **(B)** Colony expansions on CTT plates containing 0.3% agar and 1.5% agar, respectively. The pictures were taken after 5 days of incubation. **(C)** The developmental aggregation of the strains on TPM medium. The pictures were taken after 72 h of incubation. The bottom of the figure shows the strains used for fruiting body formation. The scale bars in **(B,C)** represent 1 mm. **(D)** The relative sporulation production of the strains on TPM medium. Error bars represent the standard deviation from three independent experiments.

Cell-to-cell self-recognition is a prerequisite for *Myxococcus* strains to perform multicellular social behavior, such as the formation of fruiting bodies and social motility (S-motility). The nine independent SI mutants from DK1622 formed incompatible colonies with their parent strain, as well as with each other, but these SI mutants each merged colonies by themselves. In monocultures, the SI mutants had variable growth abilities (Figure 2A). Compared with the wild-type strain, some mutants, such as SI01, SI03, SI04, and SI11, produced more than 10 times fewer colony-forming units (CFUs) after 48-h incubation. In contrast, the CFUs of some mutants, such as SI09, were similar to those of the wild-type strain.

Incompatibility occurs between two swarming colonies. The cellular motility of these SI mutants should play a role in the formation of colony-merger incompatibility. Both type IV pili (TFP) and extracellular polysaccharides (EPS) are essential to the social behavior of *M. xanthus* cells (Wu and Kaiser, 1997; Li et al., 2003). We performed Western blot experiments with the antibodies of PilA proteins (the major component of TFP) and demonstrated that the SI mutants maintained the pilus formation ability (Figure S3A). Quantitative dye binding with Congo red and qualitative colony staining with calcofluor white experiments (Black and Yang, 2004) showed that the mutants had the same ability to produce EPS as the wild-type strain (Figures S3B,C).

We assayed the swarm sizes of the SI mutants on soft (0.3% agar) and hard (1.5% agar) CTT medium to determine the motility of *Myxococcus* cells (Shi and Zusman, 1993). The colony diameters ranged from 83 to 106% of DK1622 on the soft agar plate and from 78 to 104% of DK1622 on the hard agar plate (Figure 2B), which suggested that the mutants retained a similar swarming ability to the wild-type strain. We checked the sporulation abilities of the SI mutants on TPM medium, which contains no nutrients and is normally used for assays of the development ability of *Myxococcus* cells (Kroos et al., 1986). All of the SI mutants were able to form multicellular fruiting bodies (Figure 2C). The sporulation abilities of these SI mutants varied from 40 to 86% of the wild-type strain (Figure 2D). The results suggest that compared with their ancestral strain, most of the SI mutants had reduced growth, swarming and development abilities, and some remained unchanged in the monoculture.

### Sporulation mixing efficiency of the nine SI mutants and DK1622

**Figure 3:**
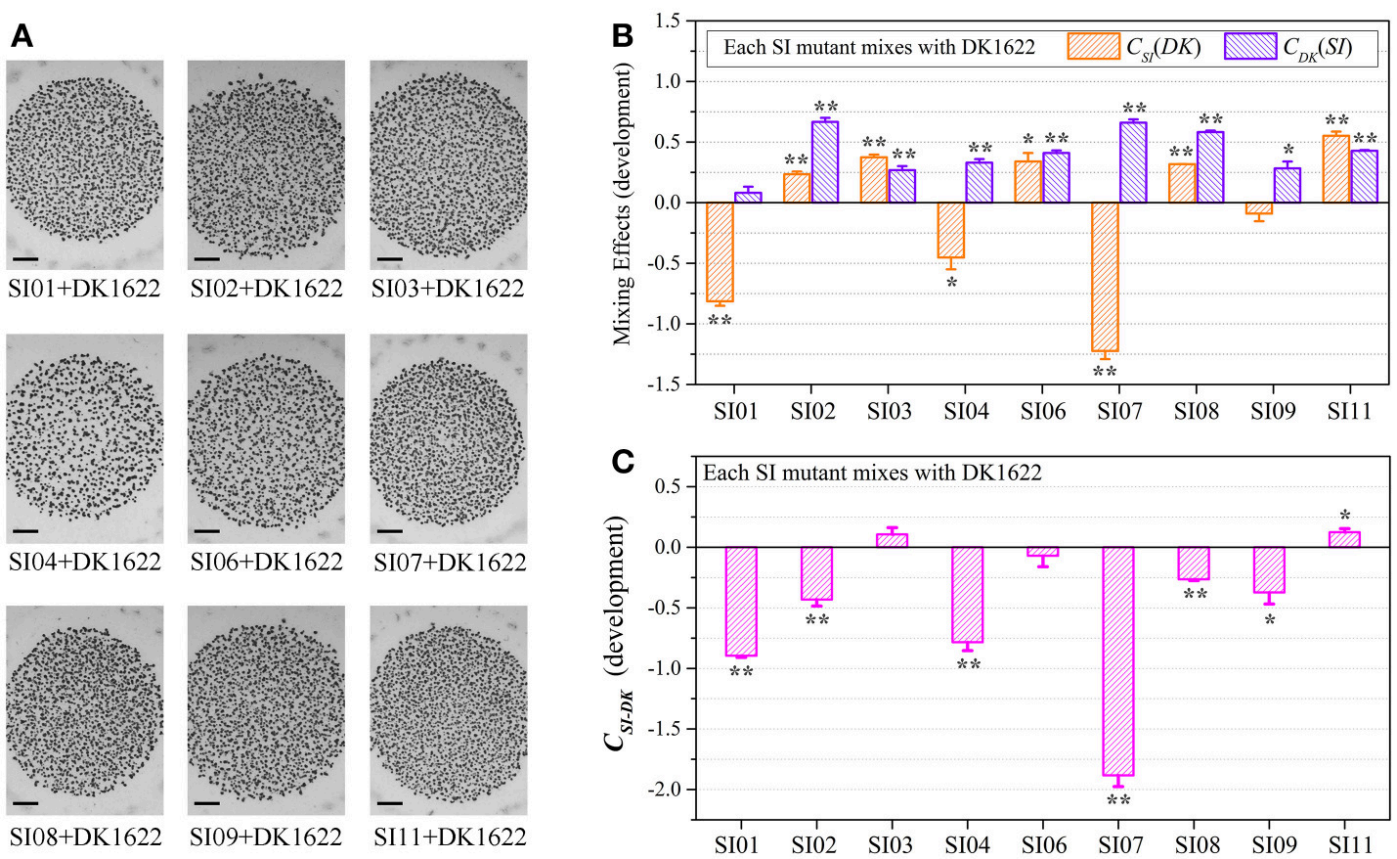
Co-development of incompatible mutants and their ancestral strain DK1622 on the TPM medium. **(A)** Morphologies of the fruiting bodies of the co-cultures. The scale bars represent 1 mm. **(B)** The log-scale difference between each strain's sporulation ability in mixture and in monoculture. **(C)** The competitive sporulation differences of co-cultured partners after eliminating the strain's sporulation difference in monoculture. Three dilutions and three replications were performed for each experiment. The error bars represent the standard deviation, and asterisks denote *p*-values for *t*-tests of differences from zero: ^*^p < 0.05, ^**^p < 0.01.

To investigate the interactions between incompatible strains, we mixed the wild-type strain with each of the nine independent SI mutants and cultivated the mixtures on the TPM development medium. The mixtures of these pairs all formed multicellular fruiting bodies, with similar morphologies to those in their monocultures (Figure 3A). After 5 days of incubation, we checked the sporulation abilities of the partners in each pair mixture based on the kanamycin-resistance characteristics of the SI mutants and the sensitiveness of DK1622. Interestingly, the sporulation abilities of the SI mutants, as well as the wild-type strain, varied in different paired mixtures (Figure S4A). We used the *W*_*SI*−*DK*_ parameter to quantify the sporulation mixing efficiency of a given SI mutant (*SI*) and DK1622 (*DK*) in the co-development experiments (Figure S4B). While the *W*_*SI*−*DK*_ values were not significantly changed for SI03 (*t*-test, *p* = 0.36) and SI11 (*t*-test, *p* = 0.11), those for the other seven SI mutants and DK1622 pairs were negative (*t*-test, *p* < 0.01). The negative values of *W*_*SI*−*DK*_ indicated that the sporulation efficiency of the SI mutant partner was lower than that of DK1622 in the mixture.

Because the sporulation abilities of the mutants varied in their monocultures, we further used the *C*_*SI*_(*DK*) or *C*_*DK*_(*SI*) parameter to deduct the effects of the strain's sporulation difference from the measurement of the partners' sporulation efficiency in the mixture (Figure 3B). A positive or negative *C*_*SI*_(*DK*) value indicates that the SI mutant sporulates more or less efficiently in the presence of DK1622 than in monoculture; it is similar to the *C*_*DK*_(*SI*) parameter. Compared with that in the monoculture, the sporulation of SI01, SI04, and SI07 was significantly reduced in the mixture with DK1622 (*t*-test, *p* = 0.02 for SI04; *p* < 0.01 for SI01 and SI07). For example, SI07 strongly decreased its sporulation efficiency when mixed for co-development with DK1622, and produced ~17 times fewer spores than in the monoculture (*C*_*SI*07_[*DK*] = −1.22). In contrast, the sporulation efficiency of SI09 was not significantly changed (*t*-test, *p* = 0.13), while the other five SI mutants sporulated more efficiently in the mixtures than in the monocultures (*t*-test, *p* = 0.01 for SI06; *p* < 0.01 for other SI mutants). Notably, except for the DK1622 and SI01 pair (*t*-test, *p* = 0.11), the DK1622 strain showed significantly greater sporulation efficiency in the co-development with each of the other SI mutants than in the monoculture (*t*-test, *p* = 0.01 for the pair with SI09; *p* < 0.01 for the others). For example, in the co-development pair of DK1622 and SI07, the sporulation efficiency of DK1622 increased and produced ~5 times more spores than in the monoculture (*C*_*DK*_[*SI07*] = 0.66).

The *C*_*SI*−*DK*_ parameter represents the sporulation difference between an SI mutant and DK1622 in the mixture after eliminating their sporulation difference in the monoculture (Figure 3C). We found that the C_*SI*−*DK*_ and *W*_*SI*−*DK*_ parameters were highly correlated (*r* = 0.98, *p* < 0.01). In the co-developed pairs of SI mutants and DK1622, most mutants exhibited significantly weaker competitive sporulation efficiencies than that of DK1622 (*t*-test, *p* = 0.02 for SI09; *p* < 0.01 for other SI mutants), while SI11 showed a slight competitive advantage over DK1622 in the mixture (C_*SI*11−*DK*_ = 0.12; *t*-test, *p* = 0.02). The sporulation efficiencies of SI03 and SI06 were not significantly affected by DK1622 in the mixtures (*t*-test, *p* = 0.08 for SI03; *p* = 0.31 for SI06).

### Competitive growth abilities between incompatible strains

We assessed the survival cells of the partner strains in the paired mixtures of SI mutants and DK1622 based on their resistance and sensitivity to the kanamycin antibiotic. The SI mutants and the wild-type strain were mixed (1:1, v/v) and incubated on a CTT plate for 48 h. In the paired mixture, the SI mutants were all outnumbered by the wild-type strain, but to different extents (Figure S5A). In the SI01-DK1622 and SI07-DK1622 mixtures, the numbers of DK1622 cells were (2.49 ± 0.32) × 10^8^ and (1.25 ± 0.35) × 10^8^, similar to the number in its monoculture, whereas the numbers of SI01 and SI07 mutants were reduced to (9.67 ± 5.69) × 10^2^ and (1.50 ± 0.70) × 10^2^ after 48 h of incubation on the CTT growth medium, respectively. The growth abilities of the other seven independent SI mutants were weakly increased or decreased, compared with those of their monocultures. For example, while the mutants weakly increased their growth abilities in the SI03-DK1622 mixture (*t*-test, *p* = 0.013) and SI11-DK1622 mixture (*t*-test, *p* < 0.01), the cell numbers of the mutant strains in the SI02-DK1622, SI06-DK1622 and SI09-DK1622 mixtures decreased.

**Figure 4:**
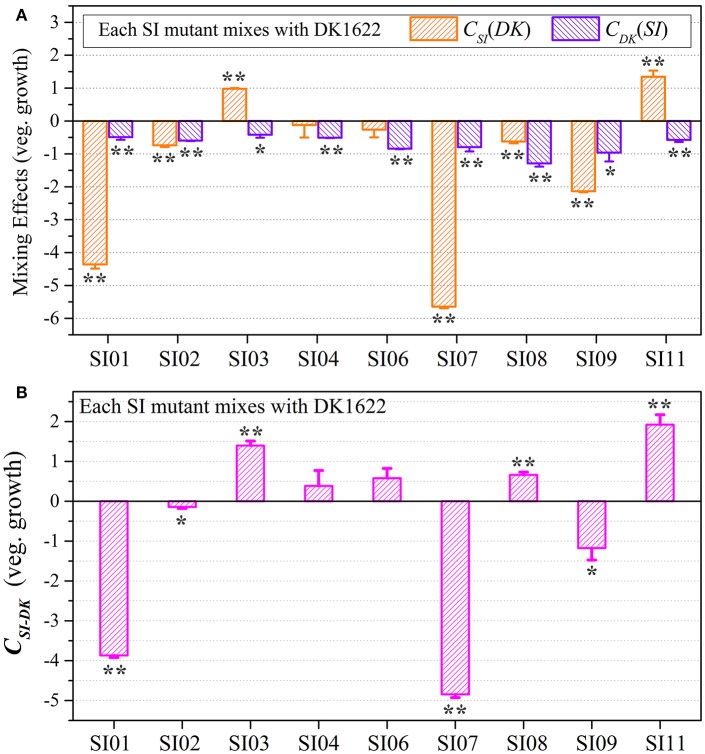
Competitive growth abilities of incompatible mutants and DK1622 in paired mixtures. **(A)** The log-scale difference between each strain's growth ability in mixture and in monoculture. **(B)** The competitive growth differences of SI mutants and DK1622 in 1:1 mixtures under vegetative growth conditions after eliminating the strain's growth difference in monoculture. Three dilutions and three replications were performed for each assay, and the error bars represent the standard deviations. Asterisks denote the *p*-values for *t*-tests of differences from zero: ^*^p < 0.05, ^**^p < 0.01.

We calculated the *W*_*SI*−*DK*_ parameter to estimate the competitive abilities between the SI mutants (*SI*) and the wild-type strain (*DK*) in mixture (Figure S5B). Except for SI11 (*t*-test, *p* = 0.29), the *W*_*SI*−*DK*_ values were all negative (*t*-test, *p* = 0.04 for SI08; *p* < 0.01 for the other SI mutants). To eliminate the effects of differences in growth ability between partners, we calculated the *C*_*SI*_(*DK*) and *C*_*DK*_(*SI*) values to estimate the strains' competitive efficiencies in their mixtures (Figure 4A). Compared with their monocultures, SI01, SI02, SI07, SI08, and SI09 showed significantly decreased growth abilities when mixed with DK1622, while the SI03 and SI11 mutants significantly increased their growth (*t*-test, *p* < 0.01). When mixed with DK1622, the growth abilities of SI04 and SI06 were not significantly different from those in their monocultures (*t*-test, *p* = 0.63 for SI04, *p* = 0.19 for SI06). However, in contrast to the sporulation increase, DK1622 significantly decreased its growth in each of the mixtures with the SI mutants (*t*-test, *p* = 0.02 for SI03, *p* = 0.03 for SI09; *p* < 0.01 for other SI mutants). The growth competition abilities between the SI mutants and the wild-type strain DK1622 in each of the mixtures, represented by the *C*_*SI*−*DK*_ values, are shown in Figure 4B. Similarly, the *C*_*SI*−*DK*_ and *W*_*SI*−*DK*_ growth parameters were strongly correlated (*r* = 0.95, *p* < 0.01). Four strains, SI01, SI02, SI07, and SI09, showed significantly weaker growth abilities than DK1622 (*t*-test, *p* = 0.02 for SI02 and SI09; *p* < 0.01 for other two), while the SI03, SI08 and SI11 strains grew significantly better than DK1622 in their co-cultures (*t*-test, *p* < 0.01). The competitive growth abilities of SI04 and SI06 in their mixtures with DK1622 were not significantly different from that of DK1622 (*t*-test, *p* = 0.23 for the SI04-DK1622 pair, *p* = 0.06 for the SI06-DK1622 pair).

**Figure 5:**
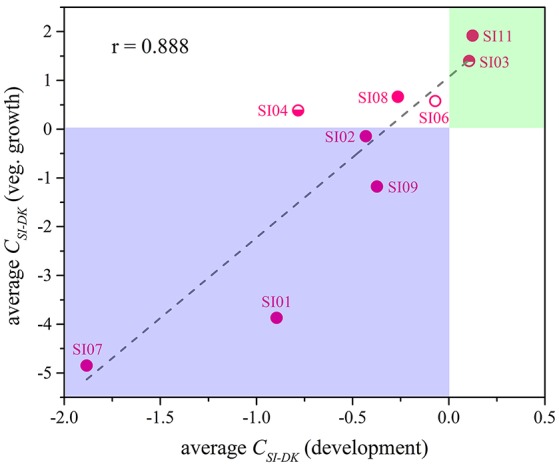
The competitive abilities between the incompatible mutants and the wild-type strain under vegetative growth and development conditions. The average C_*SI*−*DK*_ values during vegetative growth (y-Axis) strongly correlated with the values during co-development (x-Axis; *r* = 0.89, *p* < 0.01).

Although the *C*_*SI*−*DK*_ values for growth were always larger than those for sporulation, the differences in competitive growth ability between the SI mutants and DK1622 were highly correlated with their sporulation efficiency differences (*r* = 0.89, *p* < 0.01; Figure 5), thus demonstrating the consistency of competitive abilities between the SI mutants and the wild-type strain under the growth and development conditions. Overall, we can simply divide the competitive abilities between the SI mutants and DK1622 into three types: the competitive efficiency of the mutant was lower than that of DK1622 (SI01, SI02, SI07, and SI09; *C*_*SI*−*DK*_ < 0), higher than that of DK1622 (SI03 and SI11; *C*_*SI*−*DK*_ > 0), and lower for sporulation but higher for growth than that of DK1622 (SI04, SI06, and SI08). Thus, compared with the DK1622 partner, the competitive abilities of the SI mutants were improved, reduced or unchanged in the mixtures. Notably, the competitive abilities of the mutants in the mixtures were not in line with their growth or sporulation abilities in the monocultures. For example, while SI09 and DK1622 yielded similar CFUs in their monocultures, the mutant had a weaker competitive growth ability than DK1622 in the mixture. Similarly, in their monocultures, SI07 and DK1622 had similar sporulation abilities, but the mutant had markedly repressed sporulation ability when mixed with DK1622 for co-development.

**Figure 6:**
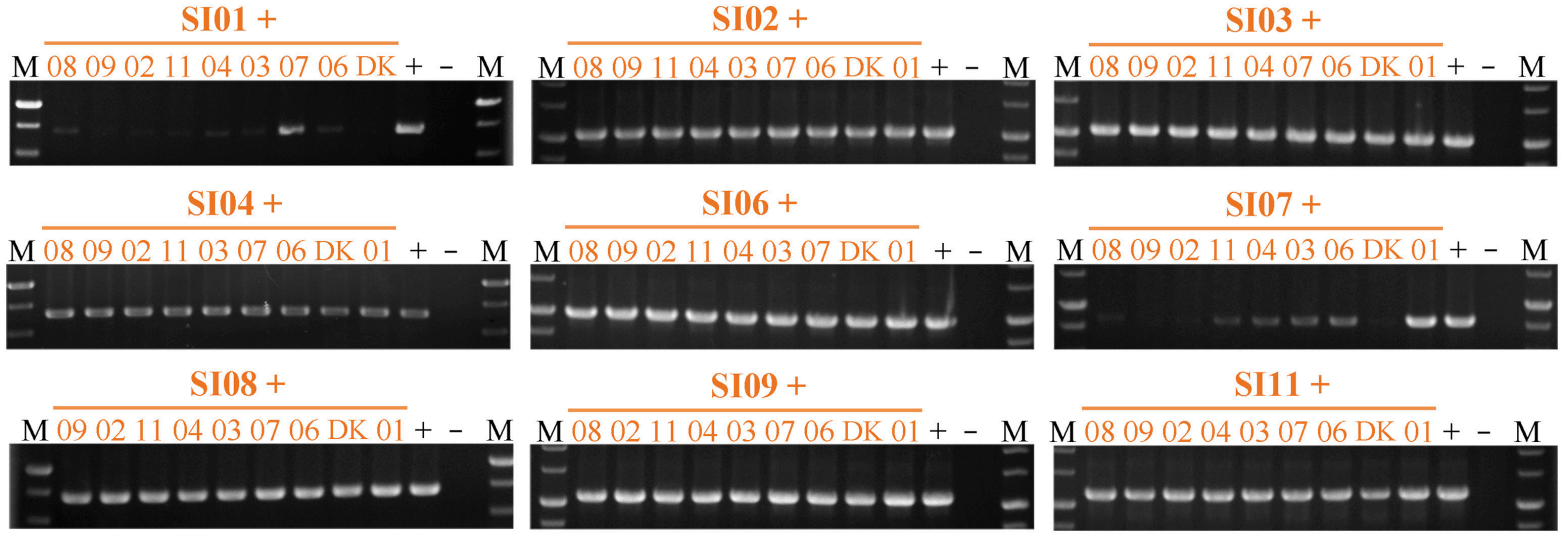
Strain-specific PCR amplification during vegetative growth to assay the presence of partners in mixed cultures among incompatible mutants. The number represents the specific SI mutant; i.e., 01 corresponds to SI01, 02 to SI02, etc. The + symbol indicates that the strain was mixed with an equal volume of TPM buffer as the positive control. The – symbol indicates the blank DK1622 control genome. M, molecular weight markers.

We also checked the survival of partners in pairwise mixtures of the nine independent SI mutants using strain-specific PCR amplifications (Figure 6 & Figure S6). All of the SI mutants except SI01 and SI07 were detectable in mixtures with any other SI mutant after 48-h incubation on the CTT growth medium. When the SI01 mutant was mix-cultivated with other SI mutants, the SI01-specific PCR amplification produced virtually no product in the mixtures with other mutants except for the band in the SI01/SI07 mixture. In the mixtures of SI07 with other SI mutants, except the SI01/SI07 pair, the SI07-specific sequence was either weakly amplified (in the mixtures with SI03, SI04, SI06, and SI11) or undetectable (in other mixtures) after 48-h incubation on the CTT growth medium. In the SI01/SI07 mixture, the SI01-specific amplification band was weak, and the SI07-specific amplification band was as bright as that of the positive control. The above results clearly indicate that the mutations in SI01 and SI07 caused the mutants to be damaged and consequently exploited by the mixture partners. When SI01 and SI07 were co-cultured, the SI07 mutant survived.

## Discussion

To date, there have been few reports on bacterial colony-merger incompatibility (Gibbs et al., 2008; Gibbs and Greenberg, 2011; Rendueles et al., 2015; Lyons et al., 2016). Diverse incompatible strains within bacterial species in small soil niches (Vos and Velicer, 2009; Stefanic et al., 2015) suggest that sibling incompatibility probably results from various genetic mechanisms. Using laboratory evolution experiments with a strain of *M. xanthus*, Rendueles et al., showed that this bacterium can easily develop into colony-merger incompatible spontaneous mutants, probably involving diverse molecular genetic mechanisms (Rendueles et al., 2015). In this study, we obtained multiple independent incompatible strains from the mutations of *M. xanthus* DK1622. These incompatible strains formed obvious colony boundaries with the ancestral strain as well as with each other, whereas the boundary did not occur between two colonies of the same strain. The mutations were scattered throughout the genome and none of them has been genetically or biochemically studied previously. The SI mutants showed varied growth abilities and retained their multicellular swarming and development abilities in their monocultures. We calculated the competitive efficiencies of SI mutants mixed with DK1622 and found that most of the mutants showed improved or reduced competitive abilities compared with the ancestral strain, but some mutants were not affected by the DK1622 pairing under either the growth or the development conditions. For example, while the growth and sporulation abilities of SI01 and SI07 were largely repressed, the growth abilities of SI04 and SI06 and the sporulation abilities of SI09 were minimally affected when mixed with DK1622. Notably, the competitive abilities of the SI mutants in mixtures were inconsistent with their growth or development abilities in monocultures.

A number of killing strategies have been shown to be involved in competitive interactions between incompatible bacterial siblings. The first mechanism to have been intensively studied is the production of small antimicrobial compounds, which act as antimicrobials or signal molecules under different conditions (Hibbing et al., 2010; Cornforth and Foster, 2013). In addition to the production of small molecule antibiotics, space competition, predation and direct poison delivery have also been reported to participate in cannibalistic interactions, such as contact-dependent inhibition and the type VI secretion system (Hayes et al., 2010; Konovalova and Sogaard-Andersen, 2011; Basler et al., 2013; Ho et al., 2014; Russell et al., 2014; Willett et al., 2015). Kin cells could be protected from the toxic proteins delivered by these pathways using specific cognate immunity proteins (Hood et al., 2010; Russell et al., 2011). Based on the survival of partners in mixture, we suggest that the systems involved in the colony-merger incompatibility of *M. xanthus* strains might include not only some kind of killing pathway for the strongly repressive effects observed in SI01 and SI07, but also some unknown mechanisms with almost no repression, as in the case of SI11. Other mechanisms that underlie the moderate interactions between other pairs could also be involved in the colony-merger incompatibilities. Some mutated genes are annotated to encode putative enzymes; for example, the mutated *MXAN_0390* gene in SI03 is annotated to encode a putative regulator of ribonuclease A, and the *MXAN_7251* gene in SI11 is annotated to encode a serine/threonine protein kinase. Some regulator genes have been shown to be involved in the kin discrimination between siblings in *B. subtilis* (Lyons et al., 2016). Additional studies are needed to identify the molecular mechanisms of kin discrimination in *M. xanthus* DK1622.

Individual organisms compete with their neighbors for shared space and food. Mixing the incompatible strains together may reduce the survival chances of one or both or even lead to the death of one or the other (Vos and Velicer, 2009; Li et al., 2013). However, incompatible *M. xanthus* strains are unable to occupy identical niches (Vos and Velicer, 2009). The competitive interactions between incompatible strains derived from *M. xanthus* DK1622 could provide a suggestion for the ecological co-existence strategy of naturally incompatible strains. The results of the current study provide evidence that the relationships between incompatible *M. xanthus* strains are diverse. Some DK1622-derived SI mutants might not survive in mixtures with DK1622, which suggests that long-term co-growth might lead to the disappearance of the inferior strains. However, separate territories, as well as the boundaries between colonies, are able to prevent the vulnerable incompatible strains from being digested by the surrounding predatory *M. xanthus* cells. We assume that after adaptive evolution in natural environments, incompatible *Myxococcus* strains localize and establish separate niche territories. The colony boundaries provide physical barriers to hinder colony mixing and avoid cannibalism, and thus allow more opportunities for separate kin groups to expand the genetic diversity within the population (Kraemer et al., 2016; Velicer and Plucain, 2016; Wielgoss et al., 2016). When the environment changes, such genetic diversity will increase the adaptation of the whole population.

In conclusion, in this study we demonstrated that multiple colony-merger incompatibilities in *M. xanthus* are associated with different genetic loci. Diverse competitive interactions between the incompatible *M. xanthus* strains indicated that the loss of compatibility might have a broad range of effects on the fitness of the mutants. The results of the incompatible *M. xanthus* mutants derived from DK1622 suggest that the relationships and ecological co-existence strategies of incompatible bacterial strains are diverse in the natural environment.

## Materials and methods

### Bacterial strains and culture conditions

The bacterial strains and plasmids used in this work are listed in Table S1. *M. xanthu*s DK1622 and its mutants were cultivated at 30°C in liquid CTT medium (Hodgkin and Kaiser, 1977). Hard CTT medium was prepared with an agar concentration of 1.5%, while the soft CTT plate contained 0.3% agar. *E. coli* strains were cultivated in lysogeny broth (LB) medium or on solid LB medium. If required, CTT and LB media were supplemented with kanamycin (Km; 40 μg ml^−1^).

### Screening for self-identification mutants

The mutants were generated using the random transposon plasmid, pMiniHimar-*lac*Z (Chavira et al., 2007). Transformants were inoculated next to each other on CTT agar plates supplemented with Km to screen for mutants in colony-merger incompatibility conditions. Colonies of mutants that could not merge with their neighbors were inoculated into liquid CTT medium containing Km and shaken at 200 rpm for 24 h. The dispersed cells were sampled, centrifuged and suspended in TPM buffer at a final concentration of 5 × 10^9^ cells/ml. Three microliter aliquots of cell suspension were pairwise inoculated on CTT plates adjacent to another cell suspension, separated by a distance of 7 mm. After 5 days of cultivation at 30°C, boundaries were observed under a SMZ100 dissection microscope. The mutants that were incompatible with their neighbors were selected for further neighboring inoculation with the wild-type strain DK1622, and the mutants that formed visually apparent boundaries with wild-type DK1622 were stored for further analyses.

### Dyeing assay of colony boundaries

The fate of boundary cells was determined using LIVE/DEAD® BacLightTM Bacterial Viability Kits, L7012 (Invitrogen, USA) following the manufacturer's fluorescence microscopy protocol with some modifications. SYTO 9 and propidium iodide dyes were mixed in equal volumes. Three microliters of the mixture were diluted in 1 ml TPM buffer and dropped onto a cover slip. The agar between two strain colonies where the boundary was formed was excised and placed upside-down on a drop of dye mixture in the dark for 15 min, and then observed under a fluorescence inverse microscope equipped with a B-2A standard filter (Nikon, Japan).

### Southern blot

To identify the single insertion site, the genomic DNAs of the strains were isolated, digested by *Sac*II, separated in a 1% agarose gel and transferred to a Hybond-N^+^ transfer membrane (Amersham Biosciences, UK). The membrane was probed with a digoxigenin (DIG)-labeled primer amplified from the *alph* gene in pMiniHimar-*lac*Z. Immunological detection was performed according to the manual provided with the DIG High Prime DNA Labeling and Detection Starter Kit I (Roche, USA).

### Mapping the locations of the insertion mutants

The insertion sites of the colony-merger incompatible mutants were localized by plasmid rescuing and sequencing (Pan et al., 2010). Briefly, the genomic DNA of mutants was extracted using the cetyltrimethyl ammonium bromide method and digested with *Sac*II. After purification by alcohol precipitation, fragments were self-circularized using T4 DNA ligase. The ligation product was transformed into the *E. coli* DH5α λpir strain. After cultivation on LB plates containing Km for ~14 h, five colonies were selected randomly for the extraction of plasmid DNA using the eZNA Plasmid Mini Kit I (Omega Bio-Tek) according to the manufacturer's instructions, and then sequenced using the primer 5′-GAA CTA TGT TGA ATA ATA AAA ACG-3′.

### Phenotypic characteristics of mutants

The mutants and the wild-type strains were assessed for motility phenotypes using standard methods (Shi and Zusman, 1993). To assay the cell-swarming capacity, aliquots (2 μl, 5 × 10^9^ cells/ml) of cells were inoculated onto CTT medium containing 1.5 or 0.3% agar. After 5 days of incubation at 30°C, we measured the sizes of swarming colonies using a Nikon D60 camera (Nikon, Japan).

Developmental ability was assessed on TPM plates using the previously described methods (Kroos et al., 1986). The strains were inoculated on CTT or CTT + Km plates for 3 days and then transferred into liquid CTT or CTT + Km medium. Cultures were centrifuged and resuspended in TPM buffer to a density of ~5 × 10^9^ cells/ml. Five microliters of the cell suspension were inoculated onto a TPM plate and incubated for 5 days. Five dots from each plate were harvested and resuspended in 100 μl of TPM buffer and then lightly sonicated to a homogenized state. After incubation for 2 h at 50°C, the cells were serially diluted, mixed with CTT containing 0.3% agar and poured into CTT and CTT + Km plates. Counting of the colonies was performed. Three dilutions and three replications were used.

### Pilus formation ability

The pilus from the strains were purified using the surface pilus preparation method described previously (Chavira et al., 2007). Pilus from DK1622 and SW504 (Δ*difA*) were prepared as positive controls, and pilus from DK10410 (Δ*pilA*) was prepared as a negative control. Western blotting was prepared using standard procedures with a 1/2,000 dilution of anti-PilA serum (Jafari et al., 2014).

### EPS production detection

For the EPS analysis, cells collected from CTT cultures were washed with TPM buffer and adjusted to a density of 5 × 10^9^ cells/ml. A 10 μl aliquot of cell suspension was spotted onto CTT plates containing 50 μg ml^−1^ of Calcofluor white M2R. After incubation at 30°C for 5 days, the plate cultures were detected under 365 nm ultraviolet light. To quantitatively analyze the EPS, a previously described method (Black and Yang, 2004) was used to bind Congo red dye to the EPS (Dana and Shimkets, 1993; Yang et al., 2000).

### Construction of *M. xanthus* mutants

Deletion mutations were generated in the wild-type strain DK1622 using standard methods (Wu and Kaiser, 1996). Upstream and downstream regions of genes were amplified, ligated together, and cloned into the plasmid pBJ113. The deletion plasmid was electroporated into DK1622, where it was integrated into the genome by homologous recombination. The strains growing on CTT plates containing Km were picked and then screened on 1% d-galactose CTT agar plates. The strains growing on CTT plates were then picked and checked by the colony PCR.

### Mixing experiments for sporulation

Strains were inoculated into the liquid CTT medium, and shaken at 30°C for 24 h to the mid-log growth phase. After being harvested by centrifugation at 8,000 × g for 5 min, the cells were resuspended in the TPM buffer to a high density of 5 × 10^9^ cells/ml. The cell suspensions of pairing strains were mixed at the ratio of 1:1 (v/v), and 10-microliter aliquots of each mixture were inoculated onto TPM plates, which were incubated for 5 days for sporulation.

To calculate the number of developed myxospores, five dots of each mixed culture were harvested and suspended in 100 μl of the TPM buffer. The suspensions were blown by the pipettor, mixed completely in a vortex mixer for 10 s, and then dispersed with a sonicator. The cell suspensions were heated at 50°C for 2 h to kill the vegetative cells. After 10-fold serial dilution, 50 microliters of the cell suspension were mixed with 2.5 ml of molten CTT soft agar and the mixtures were immediately poured onto CTT hard agar plates with Km (the growing cells were the insertion mutants) and CTT plates without the antibiotic (the growing cells included the insertion mutants and the wild-type strain DK1622). After 5 days of incubation at 30°C, the CFUs were counted to determine the sporulation abilities of strains. Three dilutions of the mixed spore suspensions and three replications for each dilution were used for the counting.

### Mixing experiments for vegetative growth

Cells were shaken in liquid CTT growth medium at 30°C for 24 h to the mid-log phase. After centrifugation, the harvested cells were resuspended in the TPM buffer and adjusted to a density of 5 × 10^9^ cells/ml. The cell suspensions of pairing strains were mixed at a 1:1 (v/v) ratio. Five-microliter aliquots of the mixed suspensions were dropped onto CTT agar medium. After 48 h of incubation at 30°C, the entire colonies were harvested, suspended in 500 μl TPM buffer and then 10-fold serially diluted in TPM buffer. To calculate the survival partner cells, cell diluents (50 μl) were mixed with 2.5 ml molten CTT soft agar and poured onto CTT hard agar plates with Km or with no added antibiotic. After 5 days of incubation at 30°C, the CFUs of the vegetative growth were counted. Three dilutions and three replications for each dilution were used for the counting.

### Competitive ability conduction

We measured the competition between different strains using the previously described method (Fiegna and Velicer, 2005) with some modifications. *N*_*SI*_ and *N*_*DK*_ represent the cell number of an SI mutant or DK1622 before pairwise mixing of the culture (*t*_0_) and the CFUs after 48 h of cultivation (*t*_2_) for vegetative growth or 5 days of culture (*t*_5_) for sporulation. The growth or sporulation ability (*D*) of an SI mutant or DK1622 in the monoculture is given as

$$\begin{array}{l} D_{SI}=N_{SI}\left(t_{n}\right)/N_{SI}\left(t_{0}\right)\\ D_{DK}=N_{DK}\left(t_{n}\right)/N_{DK}\left(t_{0}\right) \end{array}$$

The growth or sporulation ability of an SI mutant or DK1622 in the co-culture is similarly given as

$$\begin{array}{l} D_{SI}\left(DK\right)=N_{SI}\left(DK,\;t_{n}\right)/N_{SI}\left(DK,\;t_{0}\right)\\ D_{DK}\left(SI\right)=N_{DK}\left(SI,\;t_{n}\right)/N_{DK}\left(SI,\;t_{0}\right) \end{array}$$

The difference in the growth or sporulation competitive ability of an SI mutant and DK1622 in the co-culture is defined as

$$\begin{array}{l} W_{SI-DK}=\log\left(D_{SI}\left[DK\right]\right)-\log\left(D_{DK}\left[SI\right]\right) \end{array}$$

The growth or sporulation efficiency of an SI mutant and DK1622 in the mixture after eliminating the growth or sporulation difference in the monoculture is given as

$$\begin{array}{l} C_{SI}\left(DK\right)=\log\left(D_{SI}\left[DK\right]\right)-\log\left(D_{SI}\right)\\ C_{DK}\left(SI\right)=\log\left(D_{DK}\left[SI\right]\right)-\log\left(D_{DK}\right) \end{array}$$

Thus, a positive value of *C*_*SI*_(*DK*) indicates that the SI mutant grows or sporulates more efficiently in the mixture with DK1622 than in the monoculture, whereas a negative value indicates that mixing with DK1622 negatively affects the efficiency of the SI mutant. *C*_*DK*_(*SI*) has a similar meaning. After eliminating the growth or sporulation difference of each strain in the monoculture, the competitive ability between the SI mutants and the wild-type strain DK1622 in the co-culture is given as

$$\begin{array}{l} C_{SI-DK}=C_{SI}\left(DK\right)-C_{DK}\left(SI\right) \end{array}$$

### Competitive growth ability assays by strain-specific PCR

To perform the strain-specific PCR amplifications to determine the survival of partners in pairwise mixtures co-cultivated for 2 days between the nine independent SI mutants, we designed the specific primers listed in Table S2. The upstream primer was the sequence from the random transposon plasmid, pMiniHimar-*lac*Z, and was the same for all of the mutants. However, the downstream primers were specific for each mutant and were the sequence from the inserted genes.

### Statistical analysis

To examine the significance of differences, Student's *t*-tests were conducted using SPSS software. Differences were considered significant and highly significant at *p* < 0.05 and < 0.01, respectively.

## Author contributions

YG and YL designed experiments. YG, XZ, MA, XH, ZL, and XC performed experiments. ZZ, YG, XZ, and YL analyzed data. YL, YG, and ZZ wrote the paper.

### Conflict of interest statement

The authors declare that the research was conducted in the absence of any commercial or financial relationships that could be construed as a potential conflict of interest.

## Supplementary material

Video S1 Boundary formation between SI01 (left) and DK1622 (right).

Figure S1 A clear red line appeared in the colony boundary between DK1622 and SI04. The microscopic images were taken after dyeing with a mixture of SYTO 9 for live cells (in green) and propidium iodide for damaged cells or cells with an incomplete membrane (in red). The lower panels (60 × magnification) are higher magnifications of the upper image (10 × magnification). Scale bars, 0.5 and 0.1 mm.

Figure S2 The boundary formation phenotypes of the deletion mutations of the six mutated genes inserted in *M. xanthus* DK1622: the *MXAN_0049* gene in the SI01 mutant, *MXAN_0085* in SI02, *MXAN_RS36575* in SI05, *MXAN_2099* in SI07, *MXAN_RS24590* in SI08 and *MXAN_RS34540* in SI10.

Figure S3 The production of PilA proteins and EPS. **(A)** Western blotting determined the presence of extracellular type IV pili (TFP) in SI mutants, broken down from vortexed cells. SW504 (Δ*difA*), DK10410 (Δ*pilA*), and the wild-type strain DK1622 were used as controls. The yields of extracellular polysaccharides (EPS) in SI mutants were detected using Congo red binding **(B)** and calcium fluorescence staining **(C)**.

Figure S4 Sporulation abilities of mixed incompatible mutants and their ancestral strain DK1622 on the TPM medium. **(A)** The relative sporulation values of each partner in the co-development. **(B)** Differences in the sporulation abilities of co-cultured partners. Three dilutions and three replications were performed. Error bars represent standard deviations, and asterisks denote *p*-values for *t*-tests of differences from zero: ^**^*p* < 0.01.

Figure S5 Growth abilities of incompatible mutants and DK1622 in paired mixtures. **(A)** CFU numbers of paired cultured strains. SI mutants and DK1622 were mixed at ratios of 1:1 (v/v). **(B)** Differences in the growth abilities of incompatible mutants (*SI*) and DK1622 (*DK*) in 1:1 mixtures during vegetative growth. Three dilutions and three replications were performed for each assay. Error bars represent standard deviations. Asterisks denote *p*-values for *t*-tests of differences from zero: ^*^*p* < 0.05, ^**^*p* < 0.01.

Figure S6 The original gel images of the strain-specific PCR amplification products, which were analyzed by agarose gel electrophoresis and ethidium bromide staining. The number represents the specific SI mutant; i.e., 01 corresponds to SI01, 02 to SI02, etc. The + and – symbols indicate the positive and negative controls, respectively. M: molecular weight markers.

Table S1 Strains and plasmids used in this study.

Table S2 Primers used in this study.
