# Supplementary figures and images for: Competitive Interactions Between Incompatible Mutants of the Social Bacterium Myxococcus xanthus DK1622
Source: Front Microbiol. 2018 Jun 5;9:1200. doi: 10.3389/fmicb.2018.01200 (PMC5996272; doi:10.3389/fmicb.2018.01200)

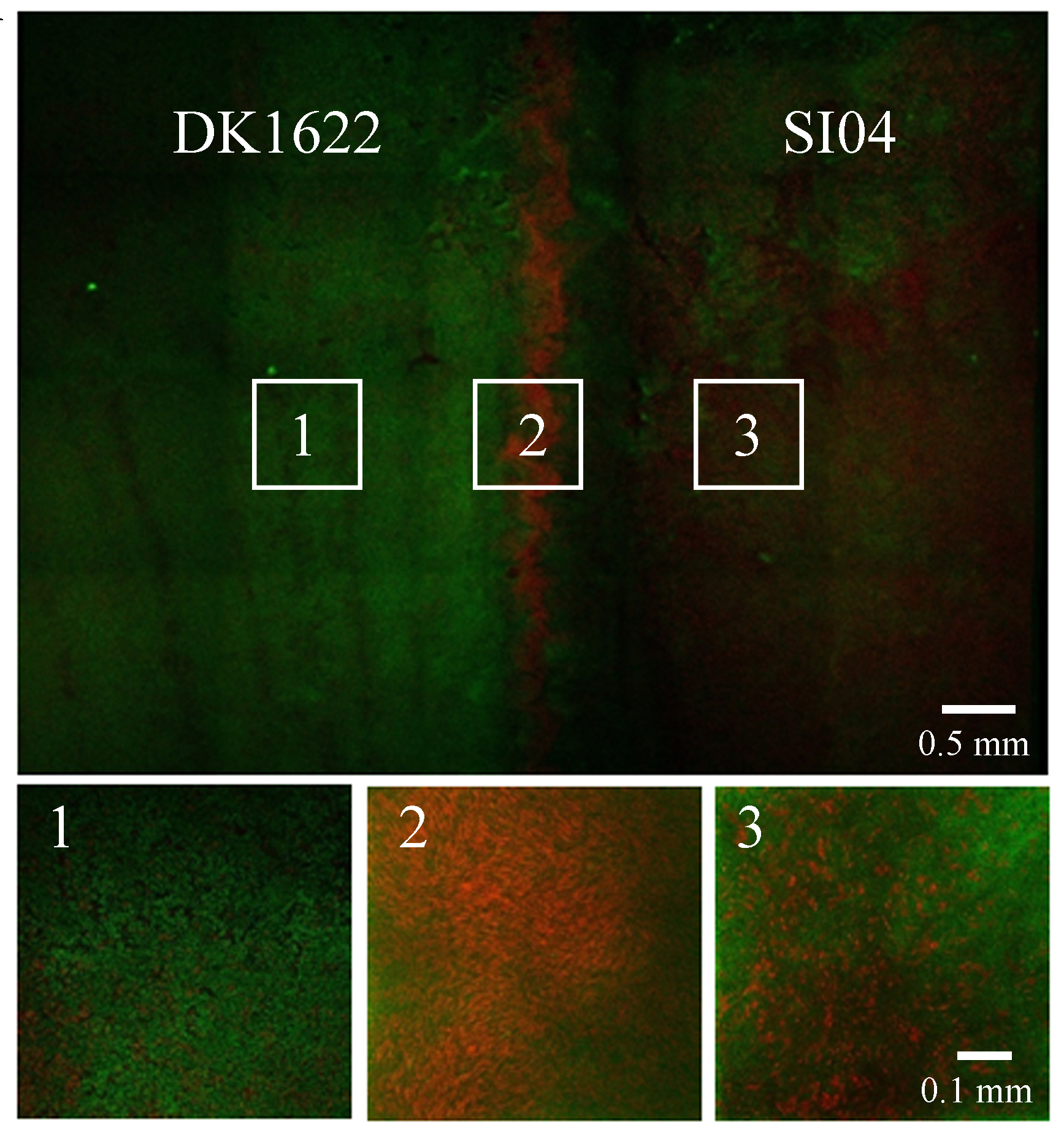

Supplement: Figure S1 — A clear red line appeared in the colony boundary between DK1622 and SI04. The microscopic images were taken after dyeing with a mixture of SYTO 9 for live cells (in green) and propidium iodide for damaged cells or cells with an incomplete membrane (in red). The lower panels (60 × magnification) are higher magnifications of the upper image (10 × magnification). Scale bars, 0.5 and 0.1 mm. [file Image_1.TIF]

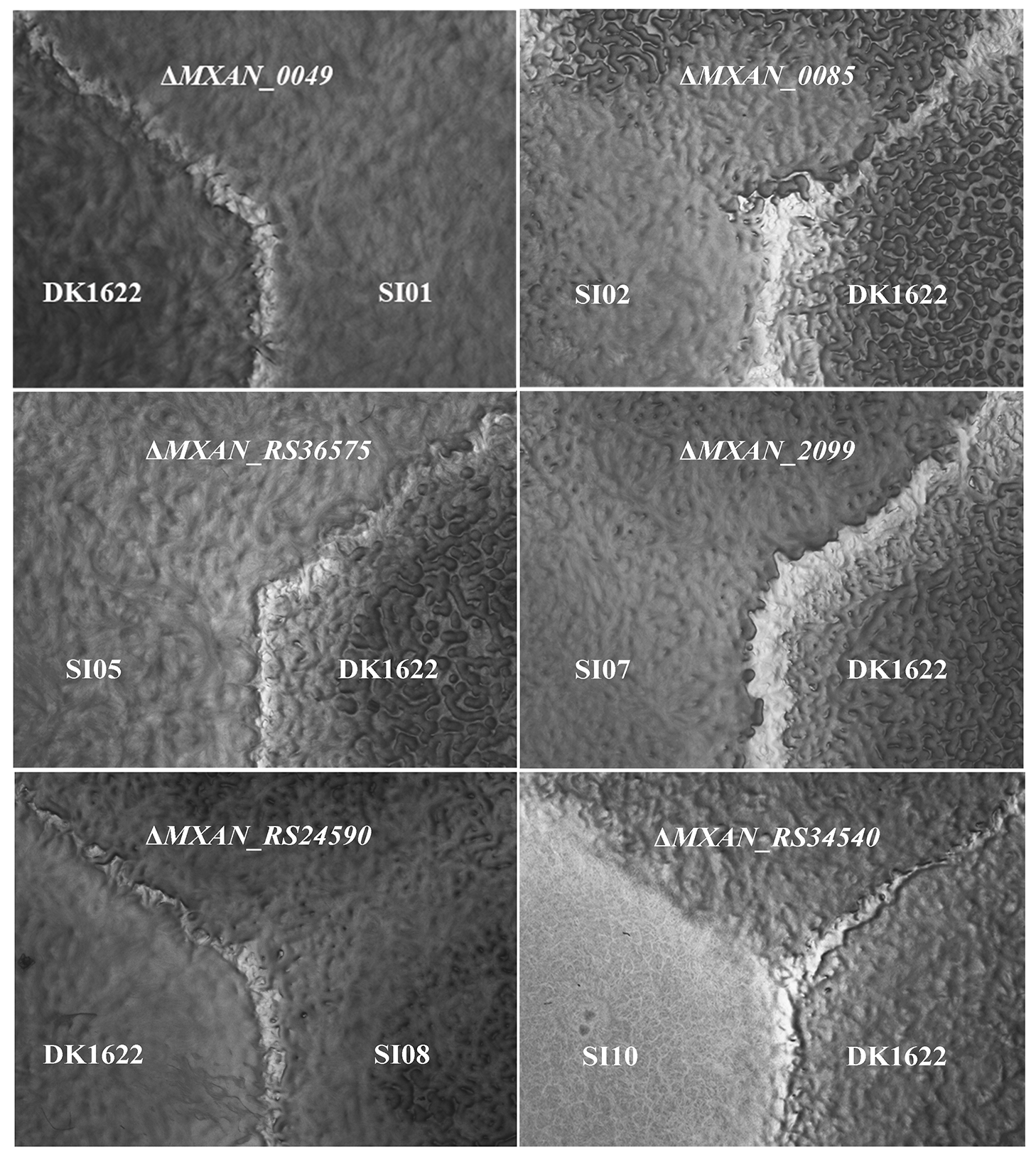

Supplement: Figure S2 — The boundary formation phenotypes of the deletion mutations of the six mutated genes inserted in M. xanthus DK1622: the MXAN_0049 gene in the SI01 mutant, MXAN_0085 in SI02, MXAN_RS36575 in SI05, MXAN_2099 in SI07, MXAN_RS24590 in SI08 and MXAN_RS34540 in SI10. [file Image_2.TIF]

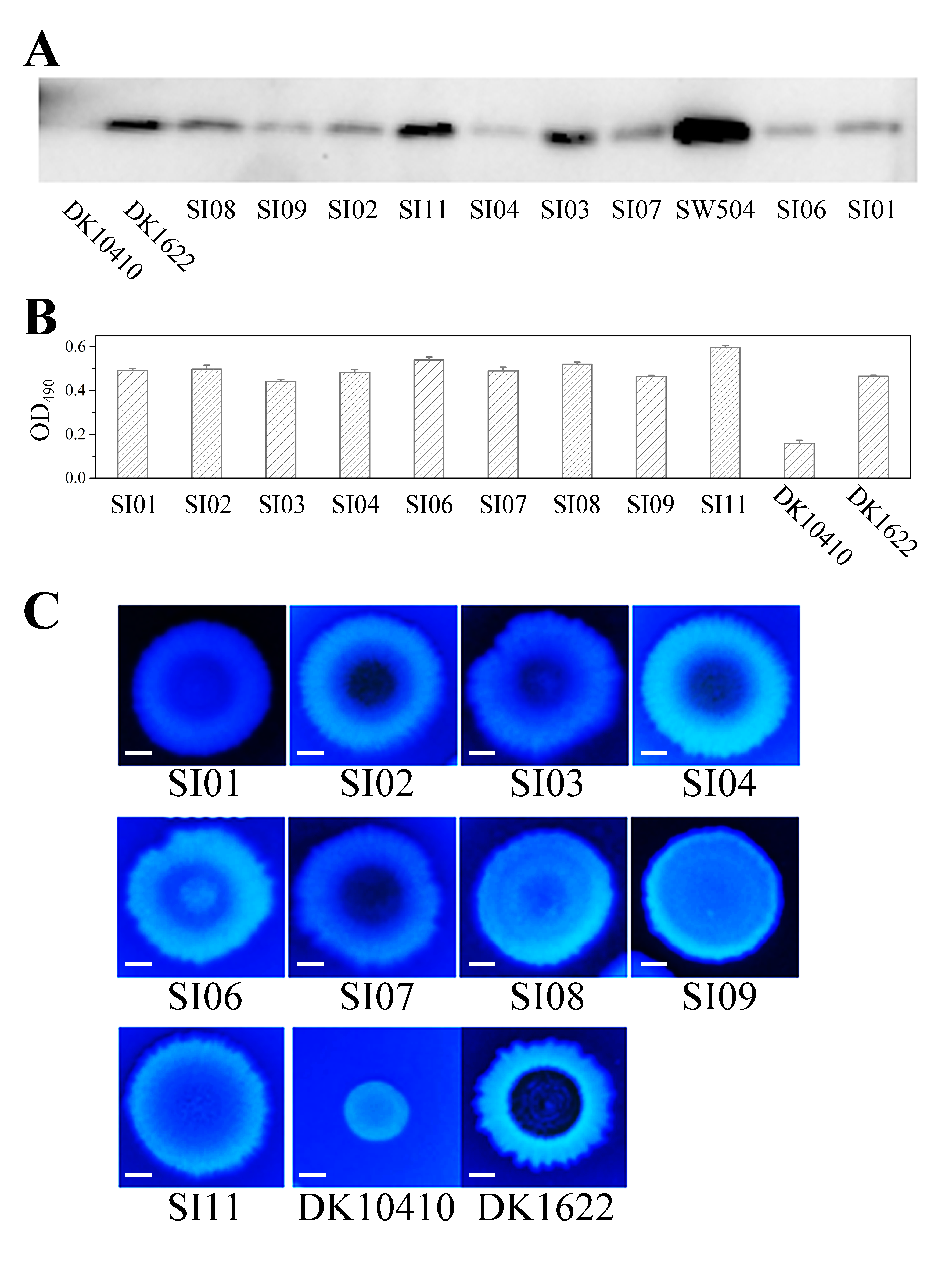

Supplement: Figure S3 — The production of PilA proteins and EPS. (A) Western blotting determined the presence of extracellular type IV pili (TFP) in SI mutants, broken down from vortexed cells. SW504 (ΔdifA), DK10410 (ΔpilA), and the wild-type strain DK1622 were used as controls. The yields of extracellular polysaccharides (EPS) in SI mutants were detected using Congo red binding (B) and calcium fluorescence staining (C). [file Image_3.TIF]

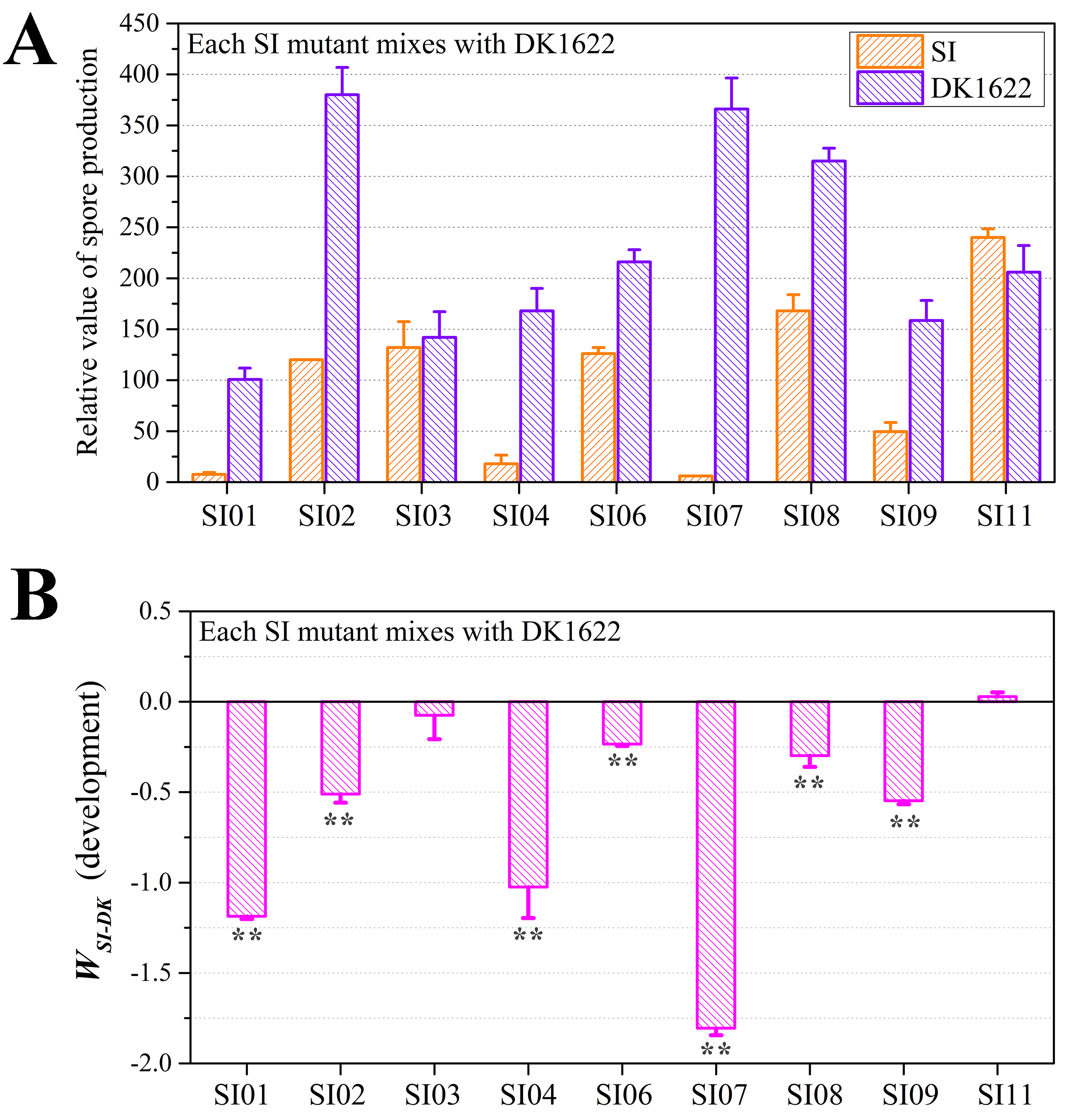

Supplement: Figure S4 — Sporulation abilities of mixed incompatible mutants and their ancestral strain DK1622 on the TPM medium. (A) The relative sporulation values of each partner in the co-development. (B) Differences in the sporulation abilities of co-cultured partners. Three dilutions and three replications were performed. Error bars represent standard deviations, and asterisks denote p-values for t-tests of differences from zero: **p < 0.01. [file Image_4.TIF]

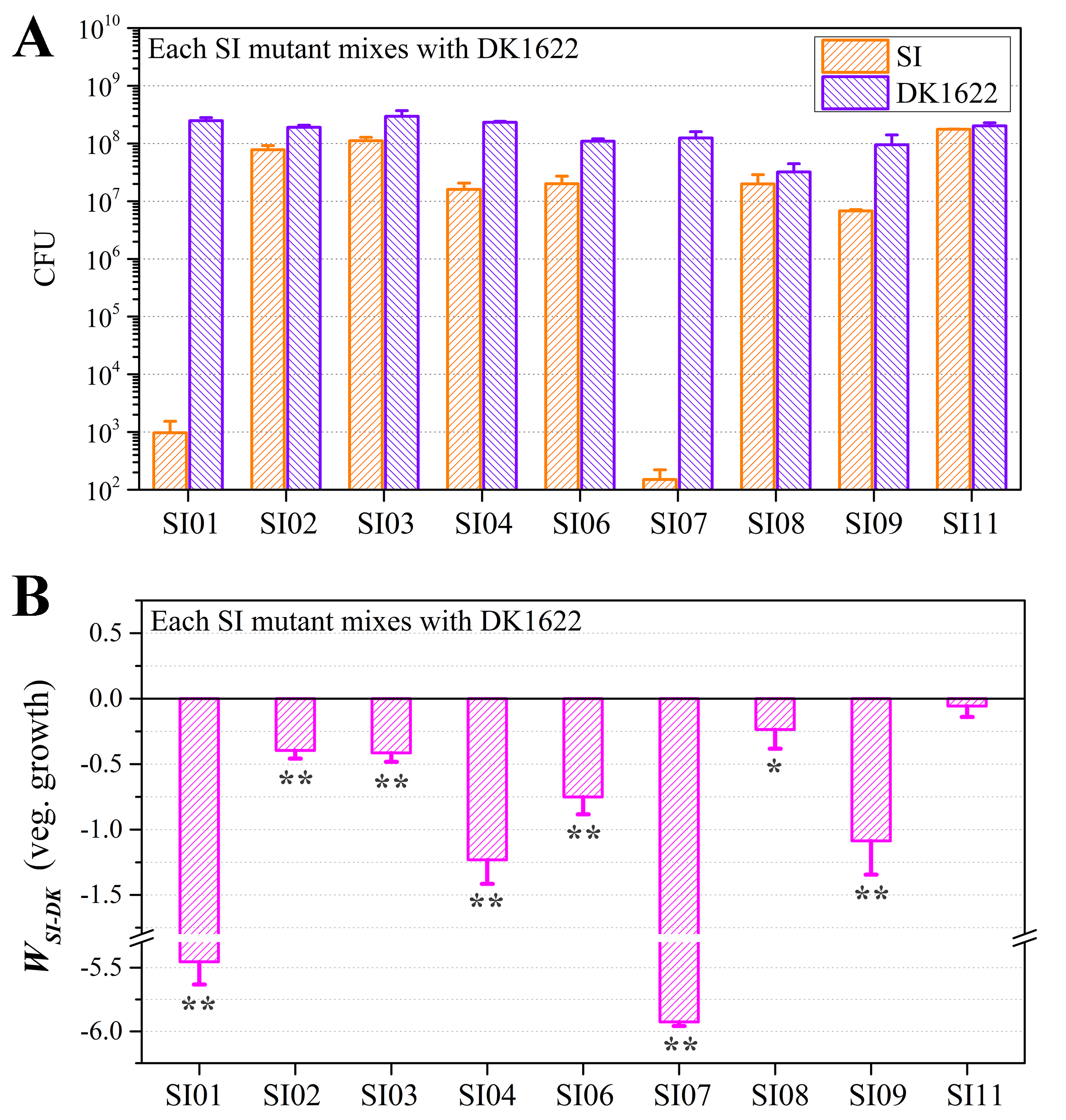

Supplement: Figure S5 — Growth abilities of incompatible mutants and DK1622 in paired mixtures. (A) CFU numbers of paired cultured strains. SI mutants and DK1622 were mixed at ratios of 1:1 (v/v). (B) Differences in the growth abilities of incompatible mutants (SI) and DK1622 (DK) in 1:1 mixtures during vegetative growth. Three dilutions and three replications were performed for each assay. Error bars represent standard deviations. Asterisks denote p-values for t-tests of differences from zero: *p < 0.05, **p < 0.01. [file Image_5.TIF]

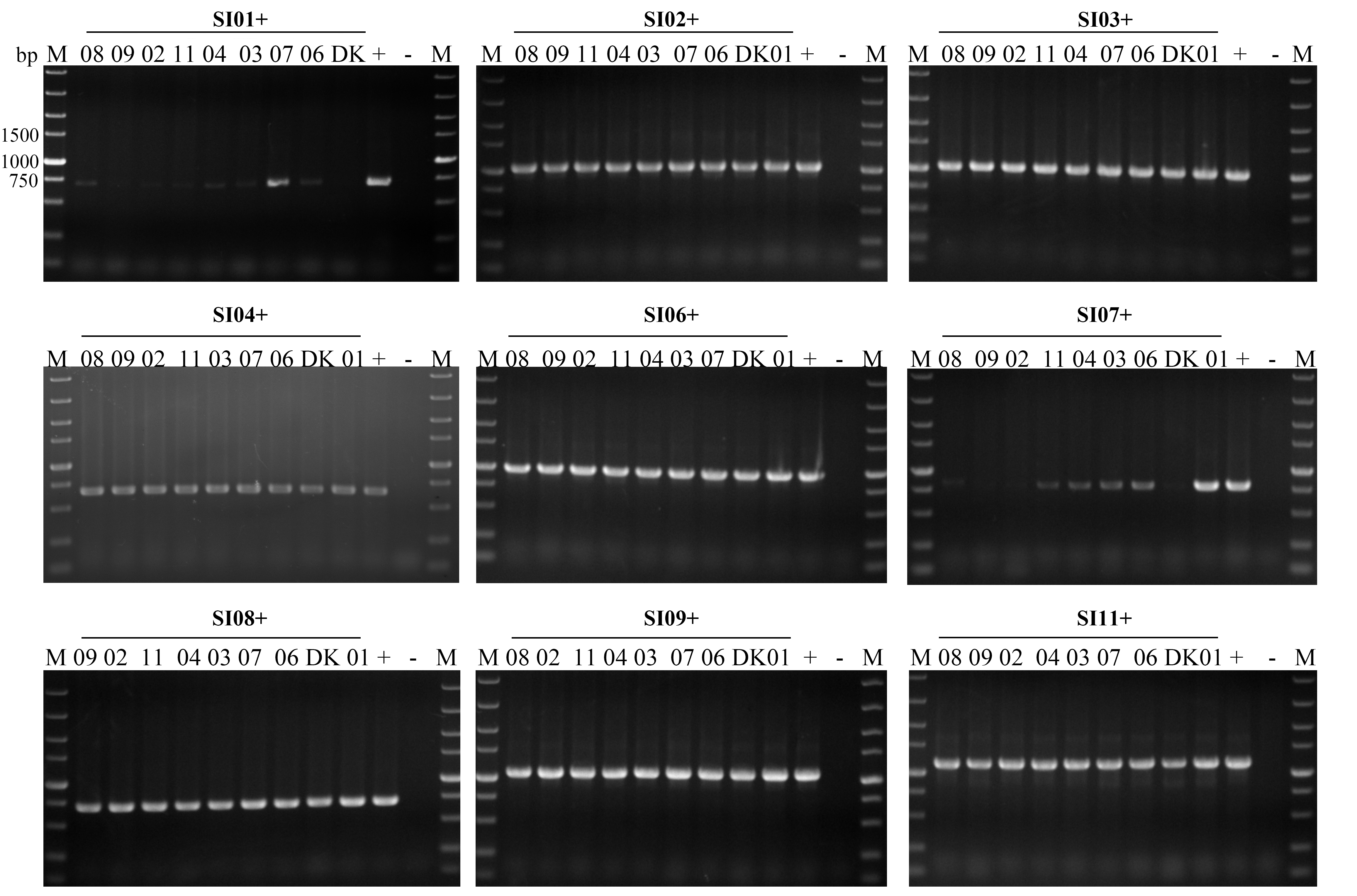

Supplement: Figure S6 — The original gel images of the strain-specific PCR amplification products, which were analyzed by agarose gel electrophoresis and ethidium bromide staining. The number represents the specific SI mutant; i.e., 01 corresponds to SI01, 02 to SI02, etc. The + and – symbols indicate the positive and negative controls, respectively. M: molecular weight markers. [file Image_6.TIF]
