# Supplementary material for: Competitive Interactions Between Incompatible Mutants of the Social Bacterium Myxococcus xanthus DK1622
Source: Front Microbiol. 2018 Jun 5;9:1200. doi: 10.3389/fmicb.2018.01200 (PMC5996272; doi:10.3389/fmicb.2018.01200)
Supplement: Table S1 — Strains and plasmids used in this study. [file Table_1.DOC]

**Table S1**. Strains and plasmids used in this study.

| Designation | Genotype or description | Source |
| --- | --- | --- |
| Strains |  |  |
| *M. xanthus* |  |  |
| DK1622 | Wild type | D. Kaiser, Stanford University |
| SI01 | insertion of *MXAN_0049*; pMiniHimar-*lac*Z; Km^r^ *lac*Z | This study |
| SI02 | insertion of *MXAN_0085*; pMiniHimar-*lac*Z; Km^r^ *lac*Z | This study |
| SI03 | insertion of *MXAN_0390*; pMiniHimar-*lac*Z; Km^r^ *lac*Z | This study |
| SI04 | insertion of *MXAN_1307*; pMiniHimar-*lac*Z; Km^r^ *lac*Z | This study |
| SI05 | insertion of *MXAN_NewI*; pMiniHimar-*lac*Z; Km^r^ *lac*Z | This study |
| SI06 | insertion of *MXAN_1599*; pMiniHimar-*lac*Z; Km^r^ *lac*Z | This study |
| SI07 | insertion of *MXAN_2099*; pMiniHimar-*lac*Z; Km^r^ *lac*Z | This study |
| SI08 | insertion of *MXAN_RS24590*; pMiniHimar-*lac*Z; Km^r^ *lac*Z | This study |
| SI09 | insertion of *MXAN_7134*; pMiniHimar-*lac*Z; Km^r^ *lac*Z | This study |
| SI10 | insertion of *MXAN_RS34540*; pMiniHimar-*lac*Z; Km^r^ *lac*Z | This study |
| SI11 | insertion of *MXAN_7251*; pMiniHimar-*lac*Z; Km^r^ *lac*Z | This study |
| DK10410 | Δ*pilA* |  |
| SW504 | Δ*difA* |  |
| DK-Km | DK1622 mx8att::*Km*, Km^r^; DK1622 + pSWU19 | This study |
| Δ*MXAN_0049* | Deletion of *MXAN_0049*, from 56489 to 57073 | This study |
| Δ*MXAN_0085* | Deletion of *MXAN_0085*, from 97026 to 97553 | This study |
| Δ*MXAN_RS36575* | Deletion of *MXAN_RS36575*, from 1533704 to 1534305 | This study |
| Δ*MXAN_2099* | Deletion of *MXAN_2099*, from 2432382 to 2432756 | This study |
| Δ*MXAN_RS24590* | Deletion of *MXAN_RS24590*, from 6337957 to 6338685 | This study |
| Δ*MXAN_RS34540* | Deletion of *MXAN_RS34540*, from 8710223 to 8710926 | This study |
| *E. coli* |  |  |
| DH5α λpir | Φ80d*lac*ZΔM15 Δ*lac*U169 *recA1 endA1*  hsdR17 *supE44 thi-1 gyrA relA1* λpir | H. B. Kaplan, University of Texas |
| XL1-Blue MR | Δ(*mcrA*)*183* Δ(*mcrCB-hsdSMR-mrr*)*173 endA1 supE44 thi-1 recA1 gyrA96 relA1 lac [F’* proAB lacI^q^ZΔM15 *Tn*10 (Tet^r^)] | Stratagene Co. |
| Plasmids |  |  |
| pMiniHimar-*lac*Z | Random transpon; Km^r^ *lac*Z | H. B. Kaplan, University of Texas |
| pSWU19 | used to integrate genes ectopically at Mx8_att_; Km^r^ |  |
| pBJ113 | Vector, galK, Km^r^ |  |

**Supplemental references**

Julien B, Kaiser AD and Garza A. 2000. Spatial control of cell differentiation in *Myxococcus xanthus*. *Proc Natl Acad Sci USA* **97**: 9098-103.

Wu SS and Kaiser D. 1995. Genetic and functional evidence that Type IV pili are required for social gliding motility in *Myxococcus xanthus*. *Mol Microbiol* **18**: 547-58.

Wu SS and Kaiser D. 1997. Regulation of expression of the *pilA* gene in *Myxococcus xanthus*. *J Bacteriol* **179**: 7748-58.

Yang Z, Geng Y, Xu D*, et al.* 1998. A new set of chemotaxis homologues is essential for *Myxococcus xanthus* social motility. *Mol Microbiol* **30**: 1123-30.
