# Supplementary material for: Competitive Interactions Between Incompatible Mutants of the Social Bacterium Myxococcus xanthus DK1622
Source: Front Microbiol. 2018 Jun 5;9:1200. doi: 10.3389/fmicb.2018.01200 (PMC5996272; doi:10.3389/fmicb.2018.01200)
Supplement: Table S2 — Primers used in this study. [file Table_2.DOC]

**Table S2. Primers used in this study.**

| **Primer name** | **Sequence** (5′–3′)* |
| --- | --- |
| The primers to amplify the probe for the southern blot | |
| Aph-up | CACGACGGGCGTTCCTTG |
| Aph-down | GAGCGGCGATACCGTAAAGC |
| The upstream primer to detect SI mutants in mixed cultures | |
| Trans-up | TCTTTGTTAGACCGGGGACTTATC |
| The downstream primer to detect each SI mutants in mixed cultures | |
| SI01-down | GGAATCTCCGTGCCTGTG |
| SI02-down | GCATCGGCCTCTTGCTTTGACA |
| SI03-down | CGGATGGAATGGGCAACG |
| SI04-down | CCGCGGCTACACCTACCTCAA |
| SI06-down | CGCTGGTGCCAGTGGTGAT |
| SI07-down | CCGCGGTGCCGGCCTCATCT |
| SI08-down | GCAGCCGAGATGGAAGAT |
| SI09-down | TCGATGCAGAGCGTACTGAAGCA |
| SI11-down | TCGCTGCTTCTGCTCTCACG |
| The primers to construct the deletion plasmid | |
| KO-0049-L-up | TGAAACCTGTCAGTCCC |
| KO-0049-L-down | TCATTCGAGATTTTTCCCCATCATGTTCGAAG |
| KO-0049-R-up | CTTCGAACATGATGGGGAAAAATCTCGAATGA |
| KO-0049-R-down | GTTCAGGCACCATACAA |
| KO-0085-L-up | GGAATTCTGGTCGAGGTCTGATGAGGG |
| KO-0085-L-down | AATCTCCGTGCCTGTGGGGTGATGAACAAACGTATAATAGCGAT |
| KO-0085-R-up | ATCGCTATTATACGTTTGTTCATCACCCCACAGGCACGGAGATT |
| KO-0085-R-down | CCCAAGCTTTTTGGGCGTCCAGGCGAAC |
| KO-36575-L-up | GTATCCCTCCATCGGCTTTG |
| KO-36575-L-down | AACCCAATAGTCCATCACCTACCAAAGGCTGCCCCA |
| KO-36575-R-up | TGGGGCAGCCTTTGGTAGGTGATGGACTATTGGGTT |
| KO-36575-R-down | CAAGGACCGAGCGAAACG |
| KO-2099-L-up | CGAGCTCCTGGGATTGCGGAAGGGATA |
| KO-2099-L-down | ACACCACCCGGACTCCCACGCACGGGATGGGCTCTTG |
| KO-2099-R-up | CAAGAGCCCATCCCGTGCGTGGGAGTCCGGGTGGTGT |
| KO-2099-R-down | GCTCTAGAGAGGAGCCCTTCCCTGTTT |
| KO-24590-L-up | GCTCTAGAGACGATGTCGCTCCGAGGCT |
| KO-24590-L-down | AGCACACGAACTAGCAGCCGAGCTCGCAGTCCTCCGGACGATTAC |
| KO-24590-R-up | GTAATCGTCCGGAGGACTGCGAGCTCGGCTGCTAGTTCGTGTGCT |
| KO-24590-R-down | CCCAAGCTTTTGCAGTCCGGGCATTCG |
| KO-34540-L-up | TTCTCCTGGAACGGATTGA |
| KO-34540-L-down | TAGGAGTGGCATACGGTGTTCATCTACCGGATGGTCACGAGA |
| KO-34540-R-up | TCTCGTGACCATCCGGTAGATGAACACCGTATGCCACTCCTA |
| KO-34540-R-down | AGGAGCGGCATTCATTTT |

*Restriction sites are underlined.
